# Bridging of membrane surfaces by annexin A2

**DOI:** 10.1038/s41598-018-33044-3

**Published:** 2018-10-02

**Authors:** David Grill, Anna L. L. Matos, Wilke C. de Vries, Sergej Kudruk, Milena Heflik, Wolfgang Dörner, Henning D. Mootz, Bart Jan Ravoo, Hans-Joachim Galla, Volker Gerke

**Affiliations:** 1 Institute of Medical Biochemistry, Center for Molecular Biology of Inflammation, University of Münster, Von-Esmarch-Str. 56, D-48149 Münster, Germany; 2 Organic Chemistry Institute, University of Münster, Corrensstrasse 40, D-48149 Münster, Germany; 3 Institute of Biochemistry, University of Münster, Wilhelm-Klemm-Str. 2, D-48149 Münster, Germany

## Abstract

The protein-mediated formation of membrane contacts is a crucial event in many cellular processes ranging from the establishment of organelle contacts to the docking of vesicles to a target membrane. Annexins are Ca^2+^ regulated membrane-binding proteins implicated in providing such membrane contacts; however, the molecular basis of membrane bridging by annexins is not fully understood. We addressed this central question using annexin A2 (AnxA2) that functions in secretory vesicle exocytosis possibly by providing membrane bridges. By quantitatively analyzing membrane contact formation using a novel assay based on quartz crystal microbalance recordings, we show that monomeric AnxA2 can bridge membrane surfaces Ca^2+^ dependently. However, this activity depends on an oxidative crosslink involving a cysteine residue in the N-terminal domain and thus formation of disulfide-linked dimers. Alkylated AnxA2 in which this cysteine residue has been modified and AnxA2 mutants lacking the N-terminal domain are not capable of bridging membrane surfaces. In contrast, a heterotetrameric complex comprising two membrane binding AnxA2 subunits linked by a S100A10 dimer can provide membrane contacts irrespective of oxidation status. Thus, monomeric AnxA2 only contains one lipid binding site and AnxA2-mediated linking of membrane surfaces under non-oxidative intracellular conditions most likely requires AnxA2-S100 complex formation.

## Introduction

Cellular membranes consist of a patchwork of lipids and proteins that have an essential function in compartmentalization and barrier formation as well as in trafficking and chemical and/or physical signal transduction events^1^. Dynamic membrane processes are driven by basic physical and chemical properties of membrane lipids and those of embedded as well as associated proteins^1–3^. Peripheral membrane binding proteins can interact with specific lipids, they can induce or assist membrane domain formation and they can link membrane surfaces to cytosolic proteins or to other membrane surfaces in the course of membrane contact formation and membrane fusion. Examples of such membrane-associated proteins are Bin/Amphiphysin/Rvs/ (BAR)^4^, pleckstrin homology (PH)- and C2 domain-containing proteins, and also the Ca^2+^ regulated annexins (Anx)^5^.

All annexins are characterized by a conserved C-terminal core domain that forms a unique membrane binding module. It comprises segments of 70 amino acids that are repeated four or eight times and harbor Ca^2+^ dependent binding sites for acidic phospholipids^6,7^. The variable N-terminal domains of annexins are accessible for interaction with cytosolic factors in their membrane-bound state and in some cases such interaction partners are small dimeric EF-hand type Ca^2+^ binding proteins of the S100 family. Based on their unique structure and lipid binding properties annexins have been linked to many membrane-related events, in particular exo- and endocytotic membrane trafficking steps, membrane microdomain formation, membrane-cytoskeleton linkages and membrane fusion^5–7^.

Annexin A2 (AnxA2) is one member of the annexin family that has been shown to function in the dynamic organization of membrane microdomains, the formation of membrane-cytoskeleton and membrane-membrane contacts. It can exist in two physical states, as monomeric AnxA2 that is predominantly localized cytosolically and on early endosomes^8^ and as a heterotetrameric (AnxA2)_2_-(S100A10)_2_ complex (A2t) that is present in the subplasmalemmal region^9^. In this heterotetramer, amphipathic alpha-helices formed by the first 14 residues of each of the AnxA2 subunits bind to two identical sites in the S100A10 dimer, thus resulting in a highly symmetrical entity, in which two AnxA2 monomers are connected via a central S100A10 dimer. As a consequence of this structural organization, the A2t complex harbors two membrane-binding annexin core modules that are most likely capable of directly linking/bridging two membrane surfaces^10,11^. Such membrane linkage is thought to underlie the function of A2t in supporting exocytotic fusions of chromaffin granules and endothelial Weibel-Palade bodies with the plasma membrane^12,13^. However, the view that only heterotetrameric AnxA2-S100A10 complexes can link membrane surface is challenged by reports describing liposome and chromaffine granule aggregations that were induced by monomeric AnxA2^14^. Such property of monomeric AnxA2 could be explained by two lipid binding sites per molecule or by direct protein interactions of two AnxA2 molecules bound to opposing membranes.

To characterize the molecular basis of the AnxA2-mediated linking/bridging of membrane surfaces and to address the central question whether monomeric AnxA2 can bridge membranes, we employed purified proteins and model membranes of defined lipid composition. Specifically, we developed a novel approach based on solid supported lipid bilayers (SLBs) and online monitoring of protein and vesicle binding kinetics by quartz crystal microbalance with dissipation (QCM-D) to analyze the membrane bridging capacity of different AnxA2 derivatives. We show that monomeric AnxA2 can link membrane surfaces, however only when undergoing disulfide-mediated dimer formation. Under reducing conditions most likely met in the cytoplasm of cells, formation of the heterotetrameric AnxA2-S100A10 complex or reported interactions with other S100 proteins such as S100A4^15^ or S100A11^16^ are probably required for membrane bridging.

## Results and Discussion

### A novel QCM-D based assay to analyze protein-mediated membrane bridging

**Figure 1:**
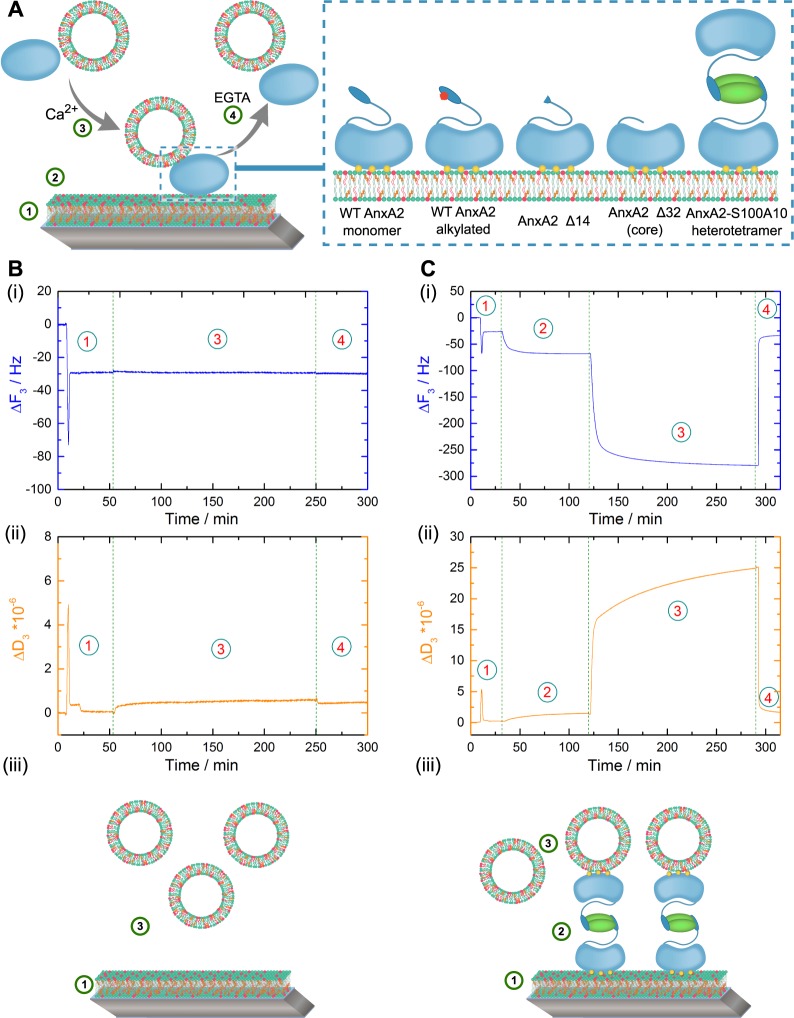
(**A**) Schematic model of the sequential lipid and protein additions in the quartz crystal microbalance with dissipation (QCM-D) setup. (1) Solid supported lipid bilayer (SLB) formation following SUV adsorption and rupture, (2) addition and adsorption of protein derivatives in the presence of 250 µM Ca^2+^ (the AnxA2 derivates used are depicted in the inset), (3) application of second membrane surface presented in the form of LUVs, (4) removal of all Ca^2+^-dependently bound protein and/or lipid by perfusion with EGTA-containing buffer. Inset: Schematic representation of the AnxA2 derivatives used in this study: (i) WT AnxA2, (ii) alkylated AnxA2, deletion mutants (iii) AnxA2 Δ32 and (iv) AnxA2 Δ14, and (v) the heterotetrameric (AnxA2)_2_-(S100A10)_2_ complex (A2t). (**B**) Addition of LUVs to a SLB layer in the absence of protein. Shown are QCM-D recordings, frequency (i) and dissipation changes (ii), revealing the formation of an SLB layer following SUV addition and rupture (step 1) and the absence of any frequency or dissipation shift upon addition of the second vesicle population in the form of LUVs (step 3). (**C**) Membrane bridging/linking by the heterotetrameric A2t complex. Time dependent frequency (i) and dissipation (ii) monitoring of SLB formation (step 1), Ca^2+^ (250 µM) dependent binding of A2t to the SLB layer containing negatively charged phospholipids and cholesterol (step 2), interaction with a secondary vesicle population (LUVs) in the presence of 250 µM Ca^2+^ (step 3) and complete reversibility of the protein and LUV adsorption by addition of Ca^2+^ chelating EGTA buffer (step 4). Models depicting the respective experimental setup are shown below the QCM-D recordings (iii). Shown are representative examples of typical experiments carried out at least n = 20 (**B**) or n = 12 (**C**) independent times with at least three different protein batches and vesicle preparations.

To directly monitor the membrane bridging/linking properties of AnxA2, we used a controllable system consisting of defined model bilayer membranes and recombinantly expressed proteins. Monomeric wild-type (WT) AnxA2 and genetically modified mutants lacking the N-terminal 14 or 32 amino acids were expressed as authentic, non-tagged proteins employing a bacterial system that faithfully executed the posttranslational N-terminal modification seen in mammalian AnxA2, *i.e*. removal of the starting methionine and acetylation of the serine residue that follows the methionine^17^. This N-terminal modification is crucially required for efficient binding of the S100A10 dimer and thus a prerequisite for reconstitution of the heterotetrameric AnxA2-S100A10 complex from its purified subunits. The truncations were chosen to either remove the amphipathic helix representing the S100A10 binding site (AnxA2 Δ14) or the entire N-terminal domain (AnxA2 Δ32). To measure the membrane bridging/linking capability of the recombinantly expressed and purified AnxA2 derivatives we developed a quantitative read-out system based on the quartz crystal microbalance with dissipation (QCM-D) technique^18^. This experimental strategy is depicted schematically in Fig. 1A. First, solid-supported lipid bilayers (SLBs) of defined lipid composition were formed on the quartz sensor (step 1)^19,20^ and then subjected to protein (AnxA2) binding (step 2). Here, the actual lipid and protein binding can be recorded online due to a change in the relative oscillation frequency of the quartz crystal that is proportional to the change in absorbed mass. Next, liposomes of defined lipid composition (LUVs) were added to the SLB-bound annexin (step 3) and protein binding to the second membrane surface presented by the LUVs was again monitored online by a change in relative oscillation frequency of the quartz crystal. Finally, all Ca^2+^-dependently bound protein and any LUVs attached via this protein were removed by Ca^2+^ chelation with ethylene glycol-bis(2-aminoethylether)-*N,N,N′,N′*-tetraacetic acid (EGTA) (step 4).

In QCM-D recordings, the correlation of negative resonance frequency shift (ΔF = F(t) − F0) with the adsorbed mass is linear for a rigid adsorbed layer as given by the Sauerbrey equation^21^:

$${\rm{\Delta }}F=\,-\,{C}_{f}\frac{{\rm{\Delta }}m}{{A}_{{\rm{q}}}}$$

However, it becomes non-linear with increasing viscoelasticity of the system. This is reflected by a change in the dissipation (ΔD = D(t) − D0) that can be taken as indicator for viscoelastic properties of the adlayer and thus the adsorbed mass during the QCM-D measurements. The dissipation (D) of the system is defined as follows:

$$D=\,\frac{{E}_{dissipated}}{2\pi {E}_{stored}}=\frac{1}{\pi f{\tau }_{0}}$$

D describes the sum of all mechanisms that dissipate energy in this oscillatory system with E_dissipated_ representing the energy dissipating during one oscillation period and E_stored_ the stored energy in the system^22,23^.

The time dependent monitoring of the formation of SLB membranes consisting of POPC/POPS/cholesterol (60:20:20) is presented in Fig. 1 B (step 1), revealing the characteristic frequency drop and thereafter, the establishment of a stable frequency shift of ΔF_3_ = −29.2+/− 0.2 Hz and a dissipation shift of ΔD_3_ = 0.45+ /− 0.09 * 10^−6^ that has been observed before for a cholesterol-containing artificial SLB membrane^24^. This drop followed by stable frequency and dissipation shifts reflects the initial adsorption of SUVs and their subsequent fusion to form a stable bilayer. Addition of LUVs (diameter of 200 nm) of the same lipid composition did not cause any change in the frequency and dissipation shifts (step 3). This control experiment was carried out in the absence of any membrane-bridging protein and indicates that no adsorption of LUVs to the SLB occurs. Next, we included an addition of the AnxA2-S100A10 heterotetramer (A2t) in step 2 of the experimental setup (Fig. 1C). This complex contains two membrane binding AnxA2 moieties and has been shown to bridge membrane surfaces by cryo-electron microscopy^25^. Addition of 50 nM A2t in the presence of 250 µM Ca^2+^ yielded frequency and dissipation shifts of ΔF_3_ = −67.5+ /− 0.05 Hz and ΔD_3_ = 1.38+ /− 0.02 * 10^−6^ (step 2) that are in line with previously reported values^26^ and indicative of high affinity protein adsorption to the SLB. After a steady state equilibrium was reached, LUVs were added in Ca^2+^-containing HBS buffer (step 3) leading to an additional frequency shift of ΔF_3_ = −279.1+ /− 0.1 Hz with a distinct dissipation shift of ΔD_3_ = 24.8+ /− 0.06 * 10^−6^. The marked frequency and dissipation changes clearly indicate the immobilization of a viscoelastic mass, *i.e*. the adsorption of buffer-filled LUVs via A2t serving as a bridge between the two membranes. Addition of 2 mM EGTA-containing buffer (step 4) resulted in a rapid increase in frequency and decrease in dissipation with the values reaching those observed for the SLB alone (step 1), thus showing that all Ca^2+^-dependently adsorbed protein and any LUVs bound via the adsorbed protein had been released. The same complete desorption upon EGTA chelation was observed for all other AnxA2 derivates used in this study (see below). Together these data indicate that A2t bound to the SLB can interact with a second membrane surface presented by the added LUVs and thus identify A2t as a membrane bridging/linking entity.

### Membrane bridging capacity of different monomeric AnxA2 derivatives

**Figure 2:**
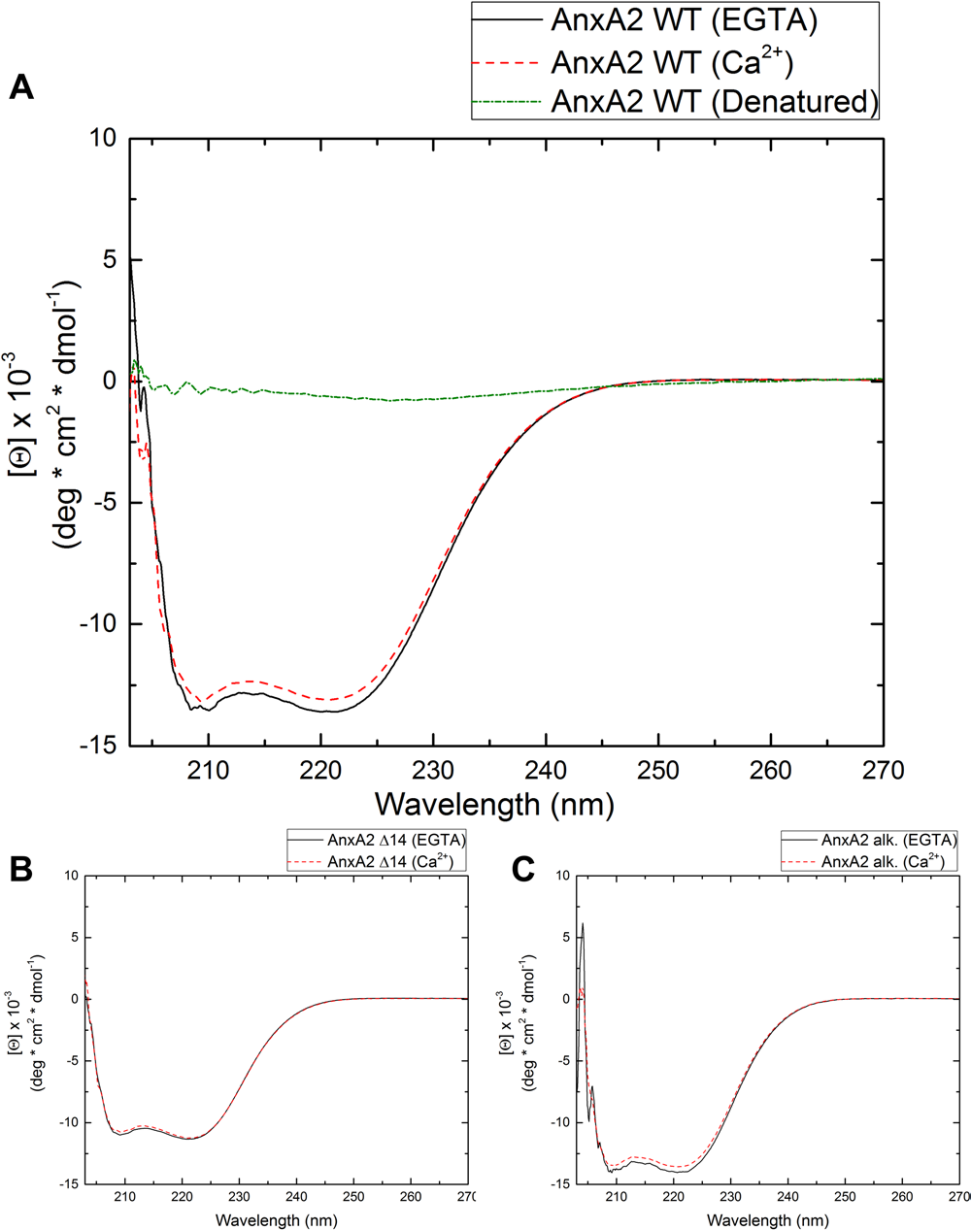
(**A**) Far-UV circular dichroism (CD) of WT AnxA2 (A), AnxA2 Δ14 (**B**) and alkylated AnxA2 (**C**) in the presence of EGTA (1 mM, black) or Ca^2+^ (1 mM, red). Measurements were performed at 25 °C in HBS (10 mM HEPES, 150 mM NaCl, pH 7.8 at 25 °C). Temperature denatured AnxA2 (green) served as unfolded control in the CD experiment. Each spectrum is an average of ten scans.

To analyze whether monomeric AnxA2 is also capable of bridging/linking membrane surfaces and to identify structural features of the protein required to mediate this activity, we employed different chemically or genetically modified AnxA2 derivatives represented schematically in Fig. 1A. Proper folding of recombinantly expressed and purified WT AnxA2 and the chemically or genetically modified derivates was verified by employing far-UV circular dichroism (CD) spectroscopy. Far-UV CD was performed in presence and absence of Ca^2+^ to verify that the different AnxA2 derivatives show the minor Ca^2+^ induced changes in mean residue ellipticity observed before for authentic AnxA2 purified from porcine tissue^27^. Recombinantly expressed WT AnxA2 as well as the modified derivatives used here show a far-UV CD dominated by α-helical secondary structure elements (Fig. 2). This is in line with the far-UV CD spectra of recombinantly expressed WT AnxA2 and different other AnxA2 mutants characterized before and the crystal structure of AnxA2 that shows a high α-helical content^28–30^. Moreover, the far-UV CD spectra reveal a small reduction of mean residue ellipticity at 222 nm in the presence of Ca^2+^, which has also been shown for AnxA2 purified from mammalian tissue^27^.

**Figure 3:**
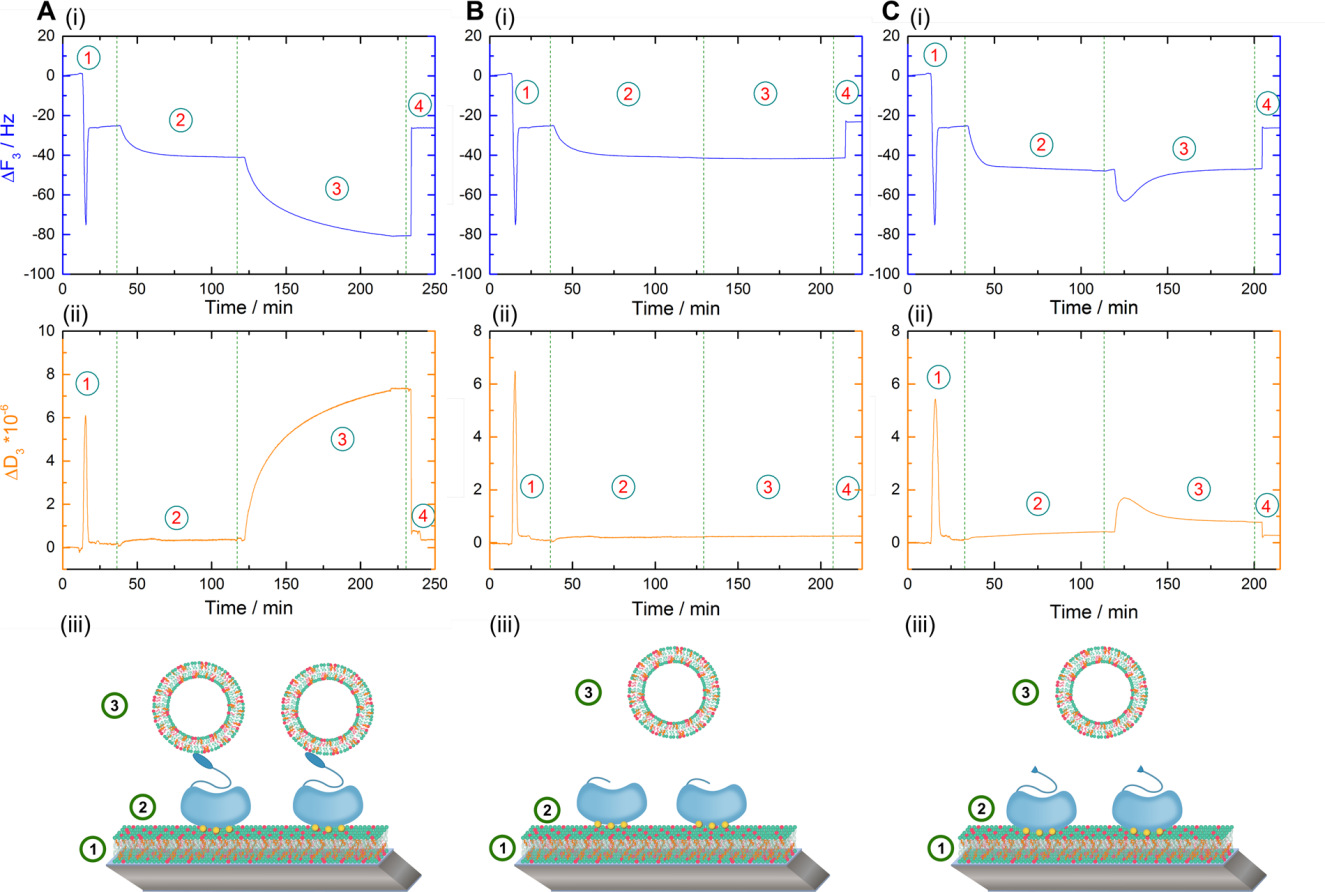
Time resolved frequency (i) and dissipation (ii) monitoring of the Ca^2+^ dependent and reversible lipid binding and membrane bridging/linking by WT AnxA2 (**A**) and the N-terminal deletion mutants AnxA2 Δ 32 (**B**) and AnxA2 Δ14 (**C**) in the QCM-D setup. Numbers illustrate the sequential addition of lipids and proteins: Step 1, SLB formation by SUV adsorption and rupture; step 2, adsorption of the respective AnxA2 derivative; step 3, secondary vesicle application in the form of LUVs; step 4, release of all Ca^2+^-dependently bound material by chelation with EGTA. Models representing the respective experimental setup are shown below the QCM-D recordings (iii). Shown are representative examples of typical experiments carried out n = 12 independent times with at least three different protein batches and vesicle preparations.

Using the above established setup, QCM-D measurements were first carried out with the inclusion of monomeric WT AnxA2 (Fig. 3A). The recordings show a behavior similar to that observed for A2t, albeit with lower and slower frequency and dissipation shifts for both, the initial protein binding to the SLB (step 2) and the subsequent adsorption of LUVs (step 3). These data suggest that monomeric AnxA2 can bridge/link membrane surfaces with the lower shifts probably due to a lower mass of AnxA2 as compared to A2t and/or a reduced number of secondary membrane binding sites.

As all known Ca^2+^-regulated membrane binding sites in annexins reside in the C-terminal core domain, we next analyzed an AnxA2 mutant (AnxA2 Δ32) lacking the unique N-terminal region and thus consisting only of the AnxA2 core domain (Fig. 3B). In contrast to monomeric WT AnxA2, this mutant, following binding to the SLB (Fig. 3B, step 2), produced no detectable frequency and dissipation shifts when the second membrane surface was offered with the administration of LUVs (Fig. 3B, step 3). Thus it appears that the N-terminal part of monomeric AnxA2 plays an important role in the bridging/linking of two membrane surfaces presented by the SLB and secondary vesicles. To further dissect the function of this N-terminal domain we generated and characterized another AnxA2 derivate that only lacked the first 14 residues known to form an amphipathic alpha-helix (AnxA2 Δ14; see above for far-UV CD spectroscopy). When employed in our QCM-D setup, this mutant showed a behavior similar to that of AnxA2 Δ32, *i.e*. a Ca^2+^-dependent adsorption to the SLB layer but no significant binding of LUVs (Fig. 3C). A minor and transient change in frequency and dissipation was observed upon application of the second membrane surface in the form of LUVs. This could reflect the presence of a low affinity membrane binding or protein interaction site in the region encompassing residues 15–32 that was removed in the AnxA2 Δ32 construct. Indirect evidence for a possible membrane interaction of the N-terminal domain of AnxA2 was indeed obtained previously by fluorescence quenching experiments involving fluorophore-labeled AnxA2. However, this interaction was observed at low pH and had not been mapped to a particular sequence in the N-terminal domain^31^. In any case, the frequency and dissipation shifts observed in our QCM-D recordings do not reflect any stable interaction as they are reverted within a few minutes of incubation (Fig. 3C, step 3). Together, these data suggest that residues 1–14 of AnxA2 are involved in mediating the binding of a second membrane surface, either by containing a second stable membrane binding site or by providing a site for protein-protein interaction and thus the formation of AnxA2 dimers.

### Membrane bridging/linking by monomeric AnxA2 is oxidation-dependent

**Figure 4:**
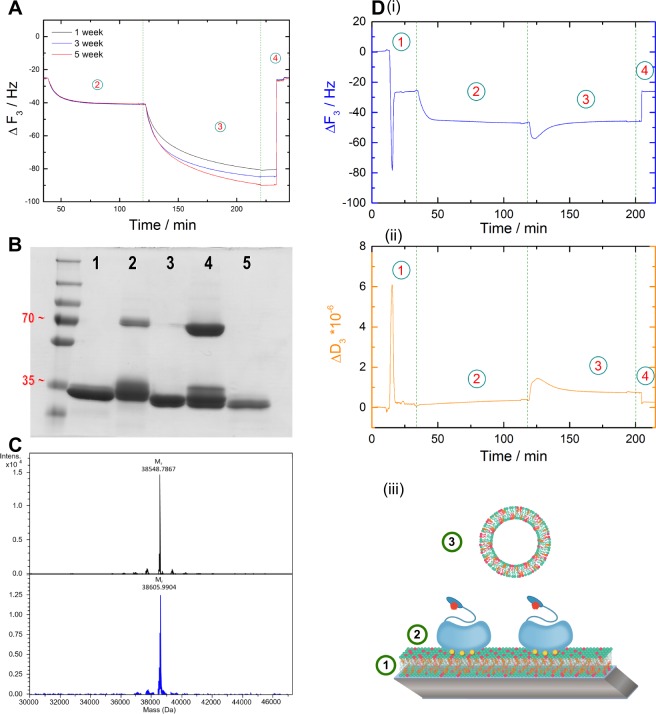
Disulfide-based dimerization of AnxA2 affects membrane bridging/linking. (**A**) Frequency and dissipation shifts monitored in the QCM-D setup depicted in Fig. 1 that are induced by WT AnxA2 following incubation of the protein at non-reducing conditions for different periods of time [week: 1 (black), 3 (blue) and 5 (red)]. (**B**) SDS-PAGE analysis of different AnxA2 derivates under non-reducing conditions: 1, alkylated WT AnxA2; 2, WT AnxA2 (oxidation state after 1 week); 3, AnxA2 Δ32; 4, WT AnxA2 (oxidation state after 5 weeks); 5, AnxA2 Δ14. PageRuler™ Plus Prestained Protein Ladder was used as a molecular weight marker. (**C**) Mass analyses of non-modified WT AnxA2 (black graph) and the alkylated derivative (blue graph). (**D**) Time-resolved frequency (i) and dissipation (ii) monitoring of the reversible, Ca^2+^ dependent binding of alkylated AnxA2. A model representing the respective experimental setup is shown below the QCM-D recordings (iii). Shown are representative examples of a typical experiment carried out n = 8 independent times with two different protein batches and vesicle preparations.

Within the 14 N-terminal residues of AnxA2, a cysteine is present at position 9. Therefore, we reasoned that the thiol group of this cysteine could potentially form a disulfide link with a neighbouring AnxA2 under oxidative conditions. This in turn would result in an AnxA2 dimer capable of bridging/linking membranes. To analyze a potential effect of AnxA2 oxidation on its membrane bridging ability, we carried out QCM-D recording with monomeric AnxA2 preparations that were kept at oxidizing conditions for different periods of time. Figure 4A shows that the time of incubation of the purified protein in the absence of reducing agents correlates with the extent of frequency and dissipation shifts occurring upon LUV addition and thus with the membrane bridging/linking capacity of the protein.

To directly address the role of potential disulfide formation through Cys-9, we aimed to chemically modify this residue by 2-iodoacetamide alkylation thereby rendering it inaccessible to cysteine crosslink. In addition to Cys-9, AnxA2 harbors three cysteine residues (Cys-133, Cys-262 and Cys-335) that are located in the core domain of the protein and are most likely inaccessible for chemical modification in the native protein. Thus 2-iodoacetamide treatment of the folded protein is likely to result in a single modification at Cys-9 in the N-terminal part^32^. To achieve this single modification we modified our purification protocol and included a 2-iodoacetamide treatment. This resulted in an efficient inhibition of oxidation-induced dimerization of the protein as revealed by SDS-PAGE under non-reducing conditions (Fig. 4B). In contrast, oxidative dimers of non-alkylated AnxA2 that increase upon storage of the protein under oxidizing conditions are readily observed in this SDS-PAGE (Fig. 4B). Furthermore, this analysis reveals that the N-terminal truncation mutants, AnxA2 Δ32 and AnxA2 Δ14, remain monomeric under oxidizing conditions further supporting the unique accessibility of Cys-9 as compared to the core domain cysteines. To further verify that 2-iodoacetamide treatment results in the modification of only one cysteine residue, we performed electrospray ionization mass spectrometry (ESI-MS) of WT and alkylated AnxA2. The deconvoluted spectrum of WT AnxA2 (Fig. 4C, upper panel) shows a major peak at 38548.7867 Da (monoisotopic), which is in good agreement with the expected monoisotopic mass of acetylated AnxA2 of 38548.7903 Da (deviation 0.09 ppm). The reaction with iodoacetamide led to a species with a major peak at 38605.9904 Da (Fig. 4C, lower panel), which corresponds to mono-alkylated and acetylated AnxA2 (theoretical monoisotopic mass of 38605.8118 Da, deviation 4.6 ppm). No double-alkylated species were observed. Thus, 2-iodoacetamide treatment can modify AnxA2 at a single cysteine residue within the N-terminal 14 residues, identifying Cys-9 as the only possible site. To probe if the alkylation of Cys-9 has any influence on the interaction of AnxA2 with negatively charged phospholipids, we first carried out liposome co-sedimentation assays. No qualitative differences were observed when liposome binding of alkylated AnxA2 was compared to that of the non-alkylated protein with both derivatives showing a Ca^2+^-dependent interaction (Figs S1 and S2, Supporting Information). Furthermore, we also performed a quantitative QCM-D-based analysis of the lipid binding of alkylated AnxA2 using SLBs containing negatively charged phospholipids and cholesterol (Fig. S3, Supporting Information). This analysis revealed interaction parameters indicative of cooperative binding characterized by a molar dissociation constants of Kd = 27.6+ /− 1.3 and a Hill coefficient of n_Hill_ = 1.96+ /− 0.11 (R^2^ = 0.996). These values are in complete agreement with those obtained earlier for non-modified WT AnxA2^26^. Based on these equivalent lipid binding parameters we next employed the mono-alkylated AnxA2 in our QCM-D-based membrane bridging/linking assay. Importantly, alkylated AnxA2 immobilized on the SLB shows no stable interaction with a second membrane surface presented by the LUVs, i.e. no additional stable frequency or dissipation shifts were obtained following LUV addition. As observed for the AnxA2 Δ14 mutant, a slight and transient change in frequency and dissipation shifts occurred immediately after LUV addition (Fig. 4D). An overview of the QCM-D data is presented in Fig. S4 (Supporting Information) which provides a statistical analysis of the normalized equilibrated frequency shifts that occurred following addition of the secondary membrane surface to the different SLB-bound AnxA2 derivatives.

Our QCM-D experiments show a strictly Ca^2+^-dependent interaction of the different AnxA2 derivatives with membranes containing negatively charged phospholipids as the addition of EGTA not only disrupts membrane bridging, i.e. removes any bound secondary vesicle (LUV), but also detaches the SLB-bound AnxA2 (Figs 1, 3 and 4). This contrasts reports showing an interaction of AnxA2 with unilamellar PS liposomes in the presence of EGTA that was particularly pronounced at lower ionic strengths of 0.1 mM NaCl^28^ and molecular dynamics simulations that suggested a direct, Ca^2+^-independent contact of lysine residues in the AnxA2 core with PS headgroups in the membrane^33^. To exclude that such potentially weaker Ca^2+^-independent interactions were missed in our online QCM-D recordings, possibly due to the relatively high flow rate of 80.4 µl/min, we carried out additional QCM-D experiments in the absence of Ca^2+^ and also reduced the flow rate to 8.4 µl/min. The results obtained under these conditions are identical to those obtained at the higher flow rates and show that no binding of oxidized WT or alkylated AnxA2 to the SLB is observed in the absence of Ca^2+^ (Fig. S5, Supporting Information) and that Ca^2+^-dependently bound AnxA2 (oxidized WT, alkylated and Δ14) is completely removed from the SLB upon addition of EGTA (Figs S6 and S7, Supporting Information). We also employed the lower flow rates to further study the minor and transient change in frequency and dissipation observed when the second membrane surface in the form of LUVs is added to SLB-bound alkylated AnxA2 or AnxA2 Δ14 (Figs 3 and 4). However, even reducing the flow rate to much lower values of 8.4 µl/min did not increase these minor QCM-D shifts supporting the view that alkylated AnxA2 and AnxA2 Δ14 lack a second high affinity membrane binding site (Fig. S7, Supporting Information).

The lack of Ca^2+^-independent binding of AnxA2 to model membranes containing roughly physiological levels of PS (20%) observed here is in line with our previous results employing SLBs and giant unilamellar vesicles of similar lipid composition^26,34,35^. However, we did observe a Ca^2+^-independent interaction of AnxA2 with endosomal membranes that was in fact mediated via the N-terminal domain^36^. We believe that this Ca^2+^-independent binding reflects a specific interaction with certain endosomal lipids not present in our model membranes or an interaction with a yet to be identified endosomal protein receptor. We have no explanation why a significant Ca^2+^-independent lipid binding of AnxA2 was observed in a recent study using unilamellar PS liposomes^28^ but think that this could be due to the different lipid compositions employed, pure PS vesicles in the study of Lopez-Rodriguez and coworkers^28^ and SLBs composed of POPC/POPS/cholesterol (60:20:20) in our QCM-D recordings. Moreover, it should be noted that the pure phospholipid mixtures used in these liposome binding studies are different from more complex phospholipid mixtures occurring in natural membranes which show, e.g., different lipid fluidities that could affect lipid-protein interactions.

**Figure 5:**
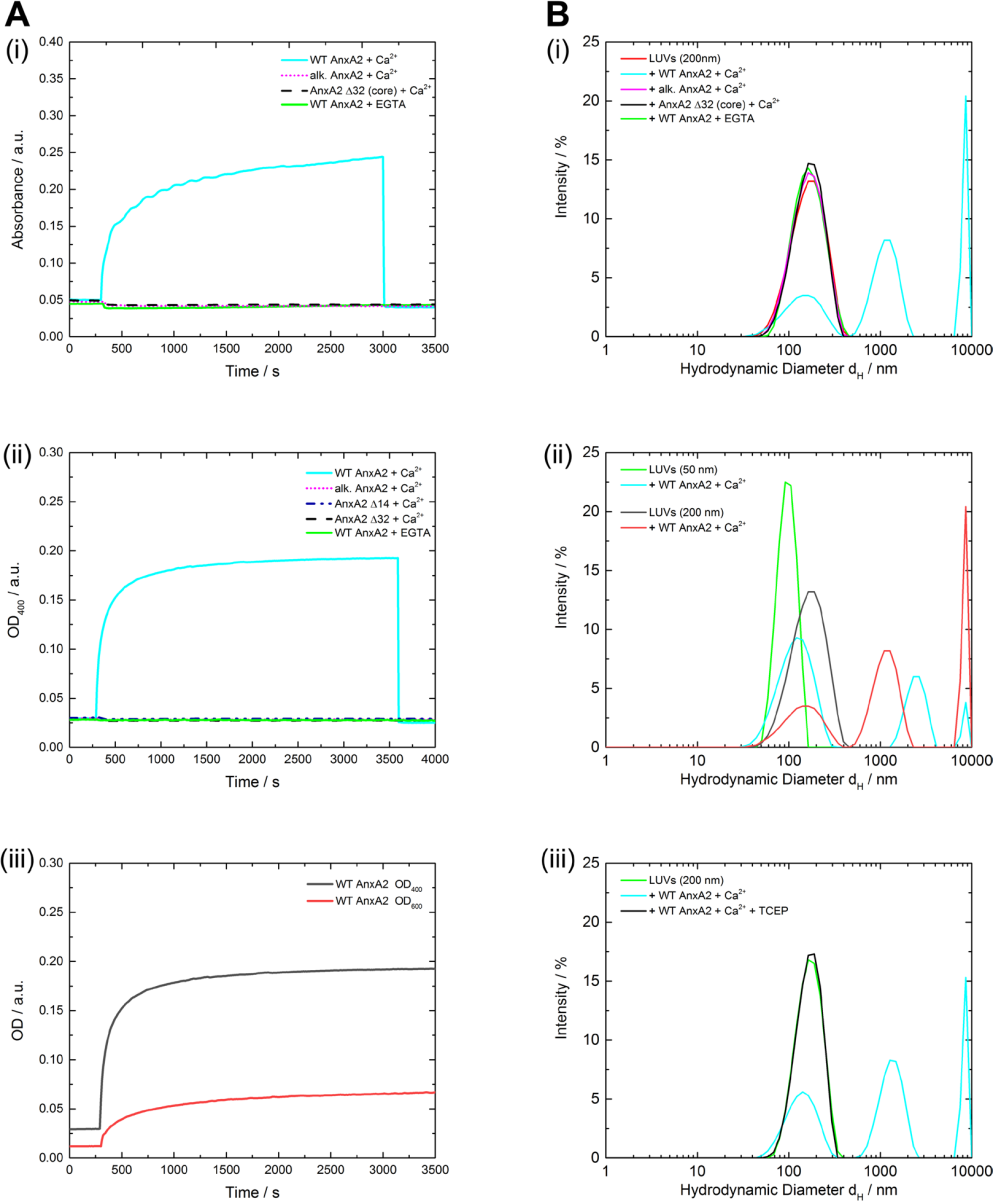
Membrane vesicle aggregation by different AnxA2 derivatives. (**A**) Time-dependent changes in optical density at OD_600_ (i) or OD_400_ (ii) during aggregation of LUVs by non-modified WT AnxA2 (oxidation state after 10 weeks), alkylated WT AnxA2 and the N-terminal truncation mutants AnxA2 Δ14 or Δ32. LUVs without addition of protein served as control. 60 µl of the respective protein sample (0.5 mg/ml) were added at *t* = 300 s to a 800 µl LUV suspension at a concentration of 0.1 mg/ml (i) or 1 mg/ml (ii) in HBS buffer supplemented with 250 µM Ca^2+^. Monitoring continued for 55 min. In (i), 650 µM EGTA in HBS buffer was added at *t* = 3000 s to revert any Ca^2+^-dependent aggregation. In (ii), EGTA was included in a parallel recording to reveal a potential Ca^2+^-independent aggregation. (iii) shows a comparison of the sensitivity of the assay at 400 and 600 nm using the same amount of LUVs (0.1 mg/ml). (**B**) Size distribution of particles present using dynamic light scattering (DLS). 60 µl of the respective AnxA2 protein (0.5 mg/ml) were added to 800 µl of a LUV suspension (1 mg/ml; size of 150 to 200 nm) in HBS containing 250 µM Ca^2+^ or 650 µM EGTA (when indicated) under non-reducing conditions. The size distribution after 60 min compared to a vesicle alone control (green) was analyzed for non-modified WT AnxA2 (oxidation state after 10 weeks), alkylated WT AnxA2 and the N-terminal truncation mutant AnxA2 Δ32 (i). In (ii), vesicle aggregation by oxidized WT AnxA2 was compared for two LUV preparations, appr. 50 or 200 nm in size using the same non-reducing conditions. A comparison of DSC measurements under oxidizing and reducing conditions is shown in (iii) where TCEP (50 mM) was included as a reducing agent in one of the recordings. Data were evaluated using a CONTIN algorithm. Graphics shown are representative example of a typical measurement. Optical density measurements were performed two times with different batches of proteins and DLS measurements were carried out at least three time with two different batches of proteins and LUVs.

To analyze membrane bridging/linking properties of the different AnxA2 derivatives by independent approaches we also performed LUV aggregation experiments. Aggregation of the LUVs following the addition of WT AnxA2, alkylated AnxA2 and the two truncation mutants AnxA2 Δ14 and Δ32, respectively, was monitored by optical density measurements at a wavelength of 600 nm (OD_600_) using identical protein and LUV concentrations. Initial equilibration of the LUV dispersion resulted in a constant OD_600_ value of around 0.05 a.u. reflecting scattering from the suspension of dispersed LUVs. Addition of non-reduced WT AnxA2 led to a significant increase of the OD600 to approximately 0.23 a.u. indicative of the formation of LUV aggregates most likely resulting from AnxA2-induced vesicle bridging/linking. In contrast, no increase of OD_600_ was observed following addition of alkylated AnxA2 or the AnxA2 Δ32 deletion mutant (Fig. 5A(i)). LUV aggregation experiments were also carried out at 10-fold lower concentrations and, to increase sensitivity of the assay at this lower LUV concentration, by measuring the optical density at OD_400_. Again, while oxidized WT AnxA2 was able to efficiently aggregate the liposomes, no aggregation activity was observed for alkylated AnxA2 and the two truncation mutants AnxA2 Δ14 and Δ32 (Fig. 5A(ii)). Moreover, oxidized WT AnxA2 was not able to aggregate LUVs in the absence of Ca^2+^. Figure 5A(iii) compares the measurements at 400 and 600 nm for the same LUV concentration. The results differ from those obtained recently by Lopez-Rodriguez and coworkers^28^ who reported that WT AnxA2 could aggregate PS vesicles even in the presence of EGTA and that the N-terminal truncation mutant AnxA2 Δ14 was also able to aggregate these vesicles. These findings are difficult to reconcile with our results, although it should be noted that a different type of vesicle population was used in the different aggregation assays. Whereas Lopez-Rodriguez *et al*.^28^ employed unilamellar vesicles composed only of PS, our LUVs contained a more complex lipid mixture (POPC/POPS/cholesterol in a ratio of 60/20/20).

Next, we employed dynamic light-scattering (DLS) to record potential protein-mediated LUV bridging/linking by changes in the hydrodynamic particle diameters. Changes were only observed under non-reducing conditions for the unmodified oxidized WT AnxA2, whereas hydrodynamic particle diameters remained constant when alkylated AnxA2 or AnxA2 Δ32 were employed (Fig. 5B(i)). To exclude that the peak in the WT AnxA2 experiment corresponding to particles with hydrodynamic diameters (<d_H_>) below 400 nm reflects the presence of smaller vesicles that could not be aggregated by oxidized WT AnxA2, we also carried out DLS experiments with smaller sized LUVs (<d_H_ > of appr. 90 nm as compared to < d_H_ > of appr. 150 nm for the experiments described above). A comparison of the results obtained for oxidized WT AnxA2 at the two LUV sizes reveals no significant differences (Fig. 5B(ii)). Aggregated particles of varied sizes including rather small sizes were observed following addition of oxidized WT AnxA2 to LUVs of higher (<d_H_ > of appr. 90 nm) and lower curvature (<d_H_ > of appr. 150 nm) and the intensity-weighted size distributions (Figs 5B(ii) and S8) do not show a selective aggregation of larger vesicles by oxidized WT AnxA2. Finally, to verify that the aggregation capacity depends on the oxidation of AnxA2, we performed DLS measurements under reducing conditions by adding the reducing agent Tris(2-carboxyethyl)phosphine (TCEP). Figure 5B(iii) reveals that WT AnxA2 shows no aggregation activity under reducing conditions. This is in line with the QCM-D and the OD_400_/OD_600_ aggregation measurements and indicates that the Ca^2+^ dependent bridging/linking of membrane surfaces by WT AnxA2 only occurs due to an oxidative disulfide crosslink involving Cys-9 and the resulting AnxA2 dimer formation.

AnxA2 is likely to remain non-oxidized and thus monomeric within the reducing environment of the cell’s cytosolic compartment. In fact, gel filtration experiments carried out with whole cell lysates have so far only revealed the existence of monomeric AnxA2 and the AnxA2-S100A10 complex^27^. Therefore, any membrane bridging/crosslinking by AnxA2 within cells is likely to require interaction with an S100 protein ligand (most likely S100A10 but possibly also S100A4 or S100A11) and thus formation of heterotetrameric AnxA2-S100 complexes. This view is supported by studies analyzing the function of AnxA2 in Ca^2+^-regulated secretory events. Both, the nicotine-induced exocytosis of chromaffin granules and the histamine- and epinephrin-evoked exocytosis of endothelial Weibel-Palade bodies require AnxA2 in the form of the AnxA2-S100A10 complex suggesting a complex-mediated membrane bridging/linking as a possible underlying mechanism^12,13^. This equivalence to cellular scenarios underscores the potential of our model system employing defined lipid bilayers and purified proteins. Therefore, we expect that our sequential QCM-D recordings of secondary vesicle binding can be applied to other peripherally binding and potentially membrane bridging/crosslinking proteins or protein complexes in the future.

## Materials and Methods

### Materials

Phospholipids, 1-palmitoyl-2-oleoyl-*sn*-glycero-3-phoshocholine (POPC), 1-palmitoyl-2-oleoyl-*sn*-glycero-3-phosho-L-serine (sodium salt) (POPS) and cholesterol were purchased from Avanti Polar Lipids Inc. (Alabaster, USA). Lipids and cholesterol were dissolved in chloroform/methanol (1:1, v/v). The HBS buffer contained 10 mM HEPES and 150 mM NaCl at pH 7.4 (20 °C) and the citrate buffer consisted of 10 mM tri-sodium citrate and 150 mM NaCl at pH 4.6 (20 °C). Solvents and other chemicals were purchased from Merck KGaA (Darmstadt, Germany), Carl Roth GmbH (Karlsruhe, Germany), Sigma-Aldrich (Munich, Germany) and Applichem (Darmstadt, Germany). Water was purified and deionized with a cartridge system from Millipore (18.2 MΩ).

## Methods

### Protein expression and purification

The human AnxA2 encoding cDNA that carries a glutamate for alanine substitution for antibody detection at amino acid position 66 was cloned into the pSE420 expression vector (psE420-AnxA2A66E^37^). Based on this plasmid, the psE420-AnxA2A66E Δ14 construct was generated by deleting nucleotides 1–42 (corresponding to amino acids 1–14) of the protein-coding region using PCR mutagenesis with the appropriate forward (5′-CATGGTTTATTCCTCCTTATTTAATCGATAC-3′) and reverse primers (5′-GATCACTCTACACCCCCAAGTGCATATGGG-3′) (Biomers, Ulm, Germany). PCR was performed in an Eppendorf Mastercycler DNA Engine Thermal Cycler PCR (Eppendorf, Hamburg, Germany) employing an annealing temperature of 55 °C for 30 s and 30 cycles. The product was purified, ligated overnight with T4 DNA ligase (Thermo Fisher Scintific, Waltham, MA) and transformed into E. coli cells. The psE420-AnxA2A66E Δ32 construct lacking nucleotides 1–96 (corresponding to amino acid 1–32) of the protein-encoding region was also obtained by deleting PCR mutagenesis as described above using (5′-GATGCTGAGCGGGATGCTTTGAACATTGAAAC-3′) as reverse primer. For the recombinant protein expression of the psE420-AnxA2A66E, psE420-AnxA2A66E-Δ14 and the psE420-AnxA2A66E-Δ32 constructs, E. coli strain DH5α was used. The cDNA encoding human S100A10 was cloned into the pKK223-3 expression vector^38^ and protein expression was performed in E. coli strain BL21(DE3) pLysS.

For protein expression, E. coli cells transformed with the respective plasmids were grown in LB medium supplemented with ampicillin at 37 °C to an optical density of 0.6 at 600 nm (OD600). Protein expression was induced by addition of isopropyl β-D-1-thiogalactopyranoside to a final concentration of 1 mM. After expression for 4 h, cells were harvested by centrifugation at 5000 x g for 10 min at 4 °C. Protein purification of all AnxA2 constructs and S100A10 was performed by diethylamioethyl- and carboxymethyl-cellulose ion exchange chromatography as described previously^9^. Reconstitution of the heterotetrameric AnxA2-S100A10 complex (A2t) using bacterially expressed AnxA2 and S100A10 was performed as described previously^17^ and involved as final step a gel filtration chromatography to separate the complex from non-complexed subunits.

Purified AnxA2 was identified using immunoblotting with mouse monoclonal anti-AnxA2 antibodies directed against an N-terminal peptide (HH7^9^) or the C-terminal core domain (BD, Purified Mouse Anti-Annexin II). Immunoblotting also employed mouse monoclonal anti-S100A10 antibodies described before^39^. Goat anti-mouse IgGs conjugated with IRDye 800CW (LI-COR Biosciences, Lincoln, USA) were used as secondary antibodies. Purified proteins were concentrated using an Amicon Ultra-4 Centrifugal Filter Unit (Merck Millipore, Darmstadt, Germany) and dialyzed against suitable buffers for the respective binding experiments. Protein concentration was determined by absorption spectroscopy using an extinction coefficient of ε_280_ = 0.7 cm^2^ mg^−1^ for all AnxA2 monomers, ε_280_ = 0.26 cm^2^ mg^−1^ for S100A10 and ε_280_ = 0.65 cm^2^ mg^−1^ for the AnxA2-S00A10 tetramer complex^27,34^.

### Alkylation of proteins

The alkylation of monomeric AnxA2 was performed with 2-iodoacetamide, commonly used for blocking thiol groups of proteins^32^. Following the reduction of cysteine residues, the alkylation by 2-iodoacetamide results in the covalent coupling of a carbamidomethyl group and prevents formation of disulfide bonds/crosslinks. For the efficient alkylation of AnxA2 the purification protocol described above was modified as follows. Lysis was performed in a lysis buffer that contained a higher amount (5 mM) of the reducing agent dithiothreitol (DTT) and an excess (250 mM) of 2-iodoacetamide as alkylation reagent. This also applied to the 2 different dialysis steps before the ion-exchange chromatography. Dialysis was performed with inclusion of DTT (5 mM) and a treatment with 2-iodoacetamide (250 mM) after the dialysis step.

### Vesicles

Lipid films of the following composition (given in molar ratios) were used: POPC/POPS/cholesterol (60:20:20). For unilamellar vesicle preparation, the respective lipids dissolved in chloroform/methanol were mixed at the given molar ratio. The organic solvent was evaporated under a stream of nitrogen at a temperature above the lipid gel-fluid phase transition. Traces of solvent were removed under vacuum for 4 h at the same temperature and lipid films were stored at 4 °C until use. Vesicles were then prepared according to established protocols^24^. Briefly, dry lipid films were suspended in the respective preparation buffer for 1 h and subjected to vortex mixing above the phase transition temperature. Small (SUVs) and large unilamellar vesicles (LUVs) were obtained by extrusion using a polycarbonate membrane^40^ of appr. 50 nm or 200 nm pore size, respectively (Avestin Liposofast). For the preparation of solid supported bilayers, SUVs were prepared in a citrate buffer. The secondary LUVs used in the QCM setup and all dynamic light scattering (DLS) and optical density (OD) aggregation measurements were prepared in HBS buffer supplemented with or without 250 µM Ca^2+^.

### Liposome co-sedimentation

The co-sedimentation assay was performed as described^41^ with some modifications. Briefly, 40 µg AnxA2 or alkylated AnxA2 were incubated with 0.32 mg of 200 nm LUVs (1.6 mg/ml) in HBS buffer supplemented with 1 mM Ca^2+^ or 1 mM EGTA in a total volume of 200 µl. After an incubation time of 1 h at 4 °C the first ultracentrifugation (uc, 96.600 × *g*, 20 min at 4 °C) step was performed. The supernatants were collected and the sediments were resuspended in 200 µl HBS supplemented with the respective additive (see above). Following incubation for 20 min at 4 °C, samples were subjected to a second uc step. The supernatants were again collected and the sediments were now resuspended in 200 µl HBS supplement with 5 mM EGTA. After an incubation time of 20 min at 4 °C and following a third uc step, the supernatants were collected and the sentiments were resuspended in 200 µl HBS containing 5 mM EGTA. Several such EGTA washing steps where carried out to completely elute all Ca^2+^-dependently bound protein. All collected fractions were then analyzed by immunoblotting with anti-AnxA2 antibodies.

### QCM-D measurements

Quartz Crystal Microbalance with Dissipation (QCM-D) measurements were performed on a Q-Sense E4 QCM-D (Q-Sense, Gothenburg, Sweden) instrument equipped with four temperature controlled flow cells in a parallel configuration connected to a four-channel peristaltic pump (Ismatec IPC, Glattbrugg, Switzerland), employing a flow rate of 80.4 µl/min. Binding analysis was performed at 20 °C in HBS buffer supplemented with 250 µM Ca^2+^. Frequency and dissipation shifts of the 3*rd* overtone resonance frequency of the quartz sensor (QSX 303, 50 nm SiO2, 4.95 MHz) were monitored. Data analysis was performed employing OriginPro v. 9.1 (OriginLab Corp.) and Hill coefficients were determined as described in^26,42^.

### Circular dichroism measurements

Far-UV circular dichroism (CD) spectra between 200 and 270 nm were recorded using a Jasco J-815 spectropolarimeter at 25 °C. All spectra were averaged over ten scans. Stock solutions of the proteins were dialyzed against 10 mM HEPES, 100 mM NaCl, pH 7.8 at 25 °C. Before the CD experiment, protein solutions were diluted to 0.4 mg/ml with the same buffer supplemented with either EGTA or CaCl_2_ to a final concentration of 1 mM. Data analysis was carried out with Spectra Manager Version 2 (JASCO) and OriginPro 9.1 (Origin).

### Measurement of optical density

Optical density (OD_600_ and OD_400_) was measured using a V 650 double-beam spectrophotometer (JASCO) at 25 °C. Samples for spectroscopic measurements were prepared in disposable 1 ml semi-micro PMMA cuvettes (BRAND) and data analysis was carried out with Spectra Manager Version 2 (JASCO) and OriginPro 9.1 (Origin). Briefly, the OD of a 800 µl LUV solution (0.1 mg/ml or 1 mg/ml in HBS buffer) was measured for 5 min before 60 µl of the AnxA2 protein solution (0.5 mg/ml in HBS buffer containing 250 µM of Ca^2+^) was added and the measurement continued for 55 min. If an increase in OD was monitored, 60 µl of EGTA (10 mM in HBS buffer) were added after 50 min to revert the Ca^2+^-dependent effects.

### Dynamic light scattering (DLS)

Measurements were carried out with a Nano ZS Zetasizer (Malvern Instruments) at 25 °C. Samples were prepared in disposable 1 mL semi-micro PMMA cuvettes (BRAND) using concentrations of 1 mg/ml of LUVs (50 nm or 200 nm) and approximately 30 µg/ml of proteins. Mixtures of LUVs and proteins were equilibrated for 60 min before each measurement. DLS measurements were performed under reducing and non-reducing conditions. TCEP (50 mM) was used as a reducing agent. Data analysis was performed with Malvern Zetasizer Software Version 7.12 (Malvern Instruments) and OriginPro 9.1. (Origin). To translate the correlation functions into size distributions a CONTIN algorithm (as implemented in Malvern research software) was applied with a quadratic weighing scheme of multimodal analysis and an automatic cut-off at a 0.01 fraction of the signal.

### Mass spectrometry

LC-MS consisted of an UltiMate™ 3000 RS (Thermo Fisher Scientific Inc., MA, USA) connected to a maXis II UHR-TOF LC-MS system (Bruker Corp., MA, USA) with a standard ESI source (Apollo, Bruker Corp., MA, USA). MS settings were as follows: Capillary voltage 4500 V, end plate offset −500 V, dry heater 180 °C, dry gas 4.0 l min^−1^, nebulizer pressure 0.4 bar. Lock mass calibration was performed using HP 1221.990364. 10 µl of a solution containing 0.1% of formic acid were injected, desalted for 5 min on a Zorbax 300SB-C3 column (3 mm × 150 mm, 3.5 µm, 300 Å) with 95% A (ultrapure H_2_O, 0.1% formic acid) and 5% B (acetonitrile containing 0.1% of formic acid) at a flow rate of 0.6 ml min^−1^ and subsequently eluted with a steep gradient of 5% to 95% B. MS data were recorded using a Control version 4.0.15.3248 and analysed with DataAnalysis 4.4 (both Bruker Daltonik GmbH, Bremen, Germany). Deconvolution was carried out using the MaxEnt algorithm and monoisotopic masses were determined by means of the SNAP algorithm, both of which implemented in DataAnalysis.

## Electronic supplementary material

Supplementary Information

## Data Availability

All data generated or analysed during this study are included in this published article (and its Supplementary Information files).
