# Supplementary material for: Bridging of membrane surfaces by annexin A2
Source: Sci Rep. 2018 Oct 2;8:14662. doi: 10.1038/s41598-018-33044-3 (PMC6168566; doi:10.1038/s41598-018-33044-3)
Supplement: Supplementary file 1 — Supplementary Information [file 41598_2018_33044_MOESM1_ESM.pdf]

Supporting Information

Bridging of membrane surfaces by annexin A2

David Grill[1], Anna L. L. Matos[1], Wilke C. de Vries[2], Sergej Kudruk[1], Milena Heflik[1], Wolfgang Dörner[3], Henning D. Mootz[3], Bart Jan Ravoo[2], Hans-Joachim Galla[3], and Volker Gerke[1*]

[1] Institute of Medical Biochemistry, Center for Molecular Biology of Inflammation, University of Münster, Von-Esmarch-Str. 56, D-48149 Münster, Germany

[2] Organic Chemistry Institute, University of Münster, Corrensstrasse 40, D-48149 Münster, Germany

[3] Institute of Biochemistry, University of Münster, Wilhelm-Klemm-Str. 2, D-48149 Münster, Germany

[*] **Correspondence:**

Volker Gerke

gerke@uni-muenster.de

Supplementary Material Online

Supplementary Figures S1 – S8

Figure S1

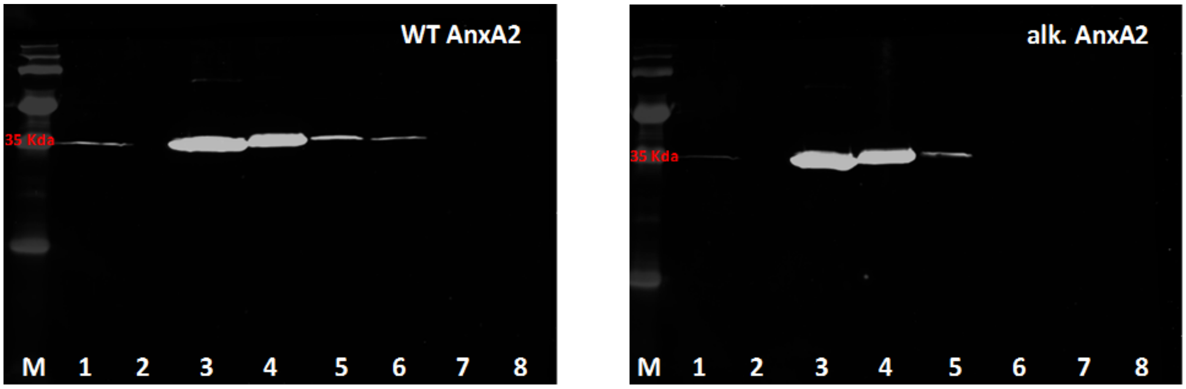

Figure S1: Liposome copelleting assay revealing a qualitative comparison of the binding of unmodified WT AnxA2 and alkylated AnxA2 to negatively charged phospholipids. Proteins were mixed with LUVs (200 nm) consisting of POPC/POPS/cholesterol in a molar ratio of (60:20:20) in HBS buffer (10 mM HEPES, 150 mM Nacl, pH 7.4 at RT) supplemented with 1 mM Ca^{2+}. Following ultracentrifugation (96.600 x *g,* 20 min at 4°C), the supernatants containing unbound protein were collected (lanes 1) and the pellets were washed with HBS plus 1 mM Ca^{2+} and centrifuged again producing a wash fraction (lanes 2). Thereafter, the liposome pellets were incubated with HBS containing 5 mM EGTA and again centrifuged to yield a supernatant containing proteins bound Ca^{2+}-dependently to the liposomes (lanes 3). Due to limiting buffer volume in the EGTA release, several additional wash step (4 in total) with the EGTA containing HBS buffer were performed and the supernatants collected (lanes 4 to 7). The final pellets containing any non-Ca^{2+}-dependently bound protein were directly resuspended in SDS PAGE sample buffer (lanes 8). All samples were analyzed by immunoblotting with specific antibodies to AnxA2. Note that unmodified and alkylated show a basically identical Ca^{2+}-dependent binding to the liposomes.

Figure S2

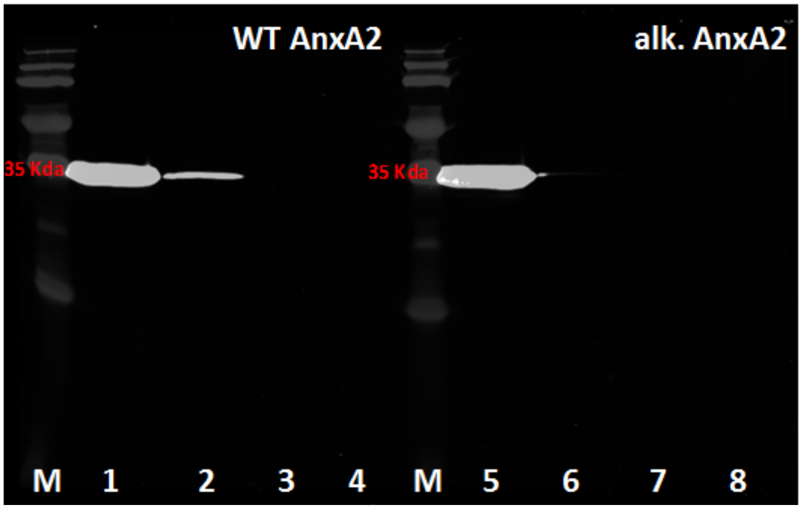

Figure S2: Liposome copelleting assay of unmodified WT and alkylated AnxA2. Proteins were mixed with LUVs (200 nm) consisting of POPC/POPS/cholesterol in a molar ratio of (60:20:20) in HBS buffer supplemented with 1 mM EGTA. Following ultracentrifugation (96.600 x g, 20 min at 4°C), the supernatants containing unbound protein were collected (lanes 1 and 5) and the pellets were washed with HBS plus 1 mM EGTA and centrifuged again producing a wash fraction (lanes 2 and 6). Thereafter, the liposome pellets were incubated with HBS containing 5 mM EGTA and again collected by centrifugation yielding the supernatant in lanes 3 and 7. The resulting pellets were directly resuspended in SDS PAGE sample buffer (lanes 4 and 8). All samples were analyzed by immunoblotting with specific antibodies to AnxA2. Note that unmodified WT and alkylated AnxA2 show a basically identical behavior, i.e. no interaction with liposomes in the absence of Ca^{2+}.

Figure S3

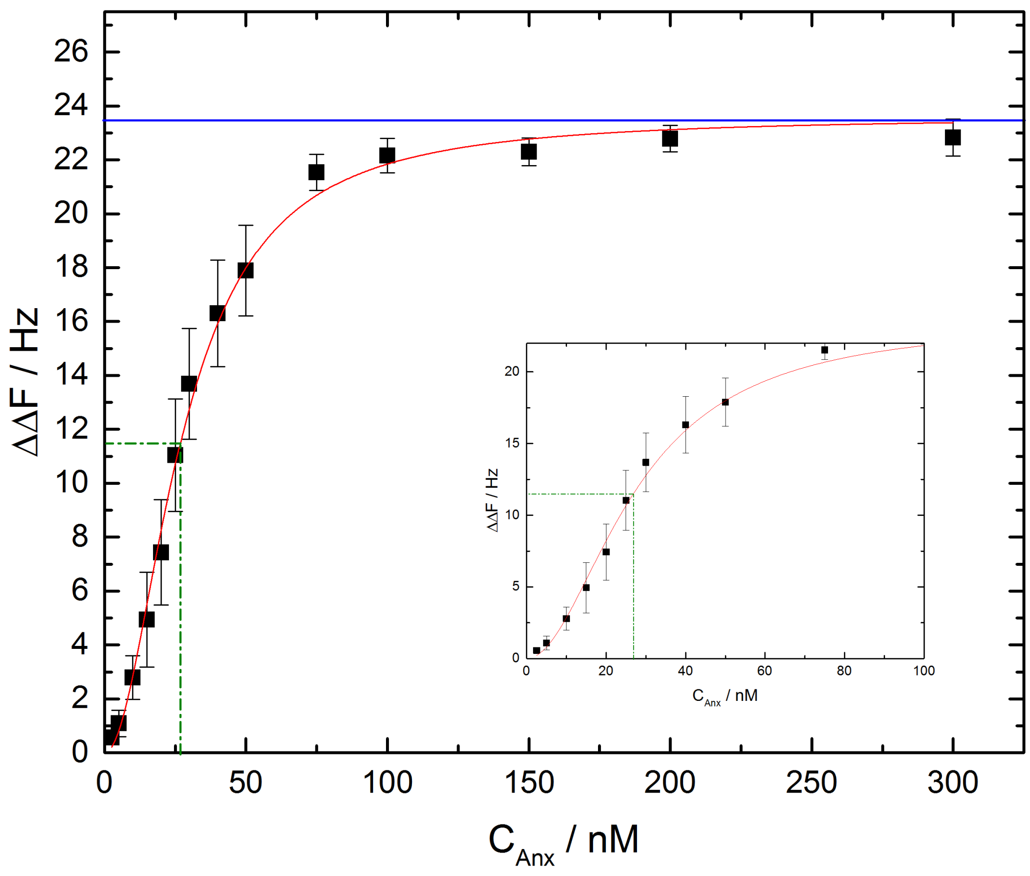

Figure S3: Adsorption isotherm of alkylated AnxA2 to SLB consisting of POPC/POPS/cholesterol (60:20:20) in the presence of 250 µM Ca^{2+} in HBS buffer. The red curve is the result of a nonlinear regression obtained of 199 equilibrium frequency shifts from 12 independent measurements and represents the Hill coefficient n for cooperativity (n = 1.96 +/- 0.11). The saturation value of the adsorption is indicated by the blue line and the corresponding green line depicts the microscopic dissociation constant $K_{d,Anx}$ = 27.6 +/- 1.3 nM at half maximum occupation in the presence of 250 µM Ca^{2+}.The insert shows the same data for lower protein concentrations at a different scale for better visualization of the sigmoidal shape.

Figure S4

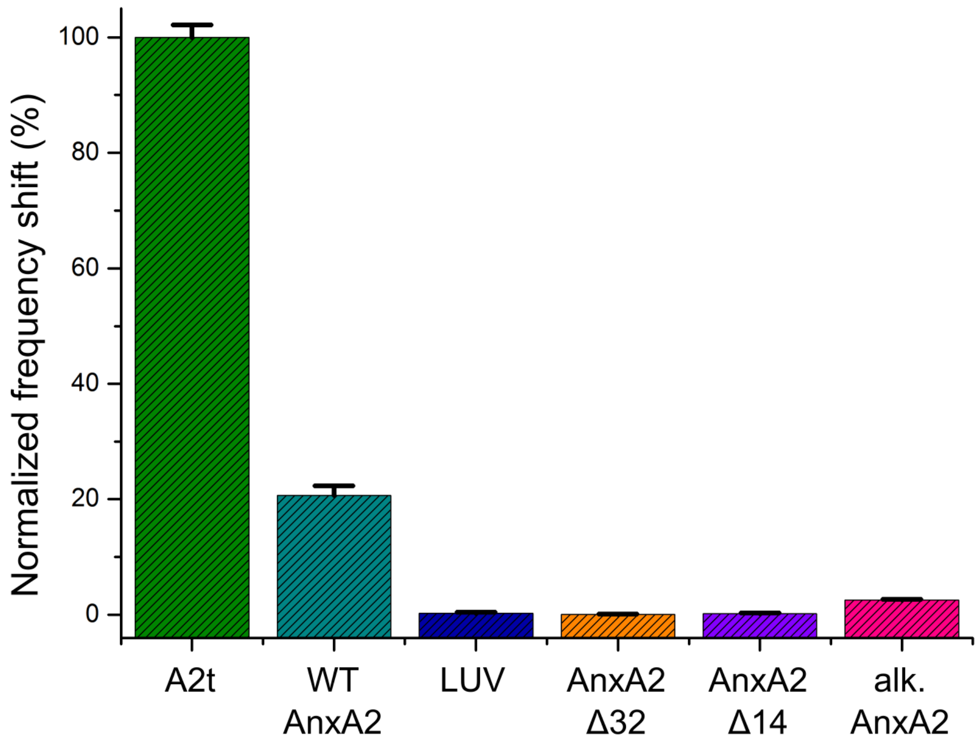

Figure S4: Statistical evaluation of the normalized equilibrated frequency shifts (in percent) that occurred upon addition of the secondary surface to the different SLB-bound AnxA2 derivatives (step three of the QCM setup). The vesicle only control is also included. Data are represented as mean ± SEM of n = 4 independent experiments.

Figure S5

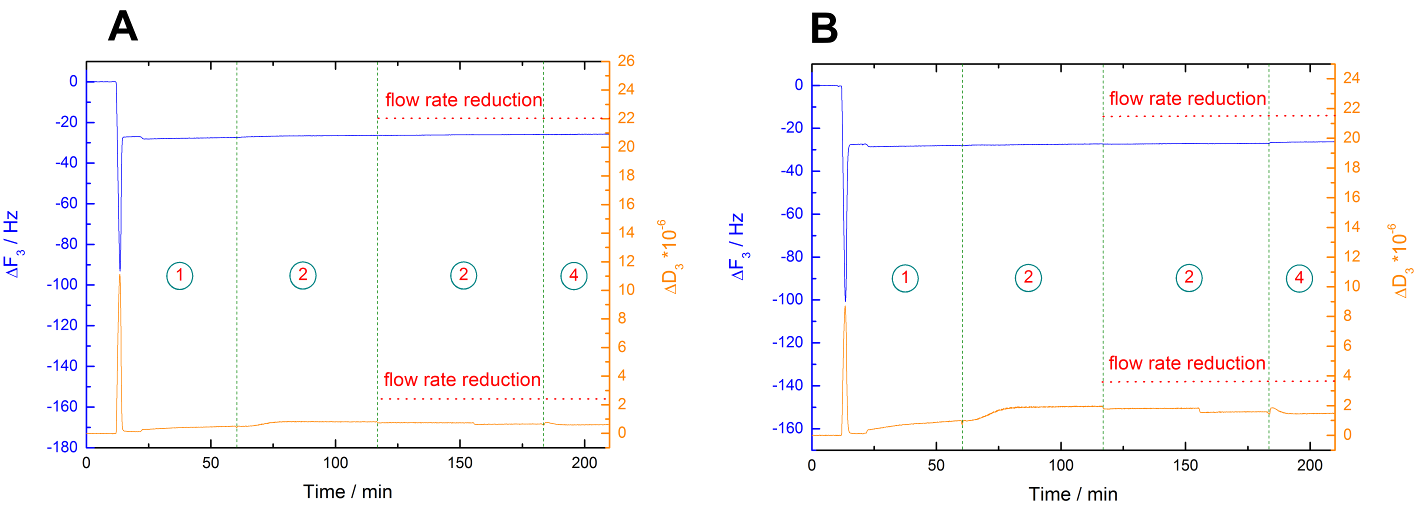

Figure S5: Time resolved frequency (blue) and dissipation (orange) monitoring in the QCM-D setup of a possible SLB interaction of unmodified WT AnxA2 (A) and alkylated AnxA2 (B) in the absence of Ca^{2+}, i.e. in HBS buffer containing 1 mM EGTA. Numbers illustrate the sequential addition of lipids and proteins: Step 1, SLB (POPC/POPS/Cholesterol 60:20:20) formation by SUV adsorption and rupture; step 2, adsorption of the respective AnxA2 derivative; step 4, release of any bound protein by additional EGTA application in the absence of protein (1 mM EGTA in HBS). The dotted red line marks a reduction in the flow rate from 80.4 µl/min to 8.4 µl/min during step 2. Shown are representative examples of a typical experiment carried out at least n = 12 times with at least three different vesicle preparations and protein batches (three independent expressions and purifications).

Figure S6

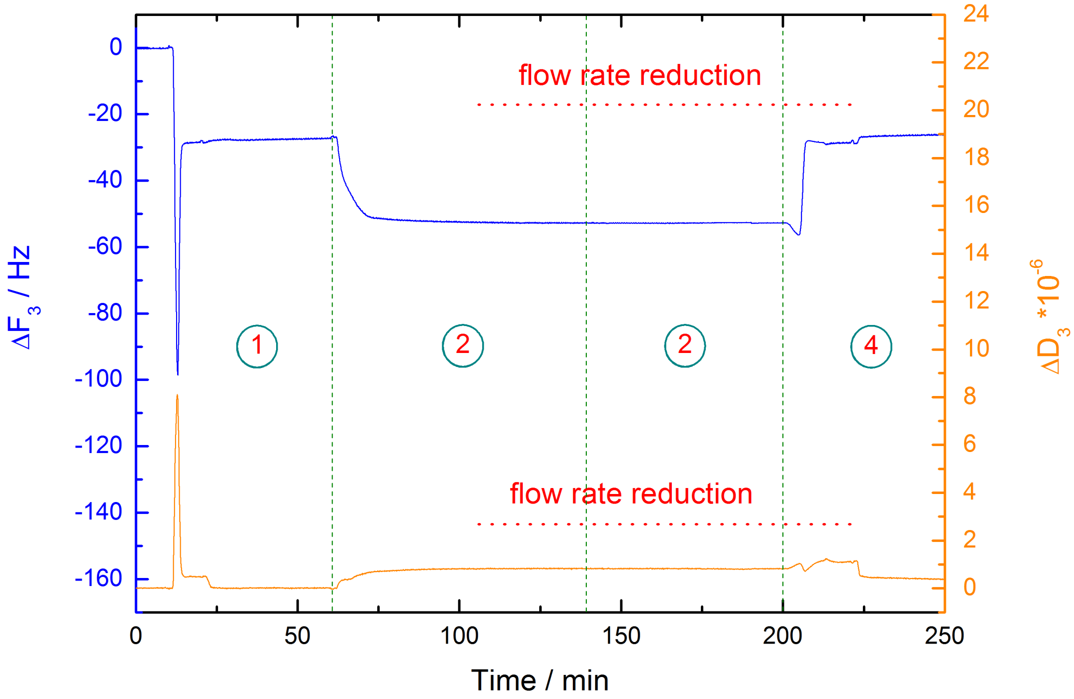

Figure S6: Binding of WT AnxA2 to solid-supported bilayers in the QCM-D setup is not affected by the flow rate. Time resolved recordings of frequency (blue) and dissipation (orange) shifts in HBS buffer supplement with 250 µM Ca^{2+}. Numbers indicate the sequential addition of lipids and proteins: Step 1, SLB formation by SUV adsorption and rupture; step 2, application of WT AnxA2; step 4, release of Ca^{2+}-dependently bound protein by chelation with EGTA. The dotted red line marks a reduction in the flow rate from 80.4 µl/min to 8.4 µl/min during step 2. Shown is a representative example of n = 4 independent measurements.

Figure S7

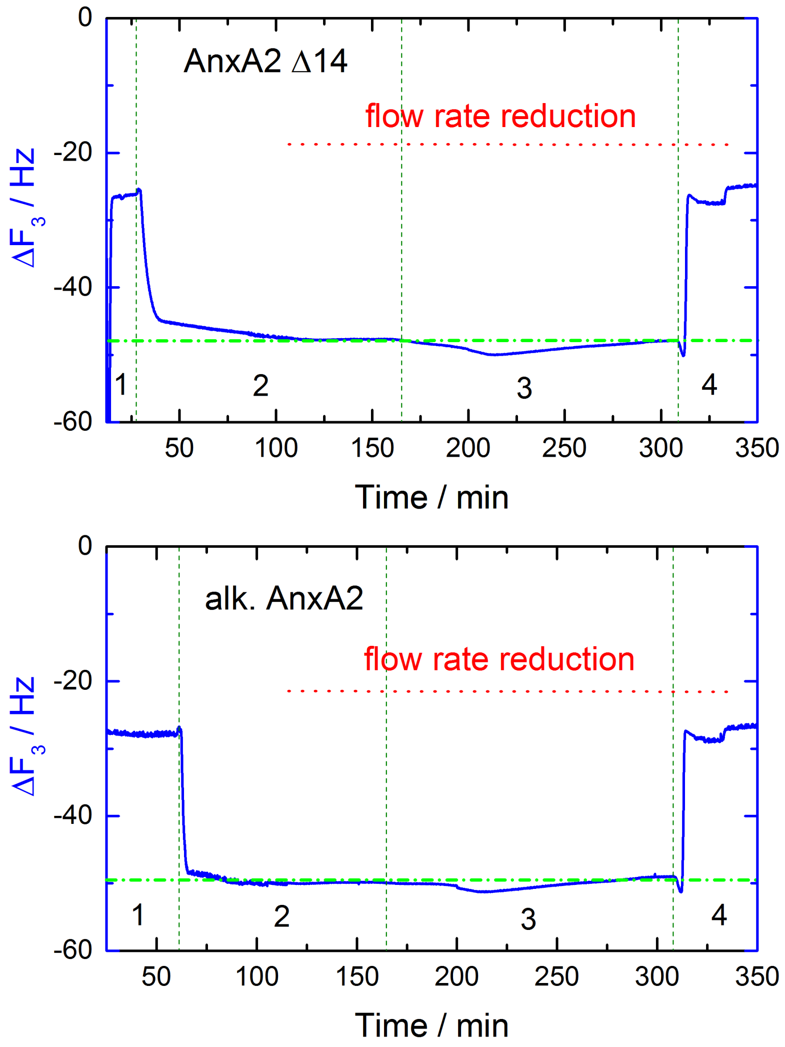

Figure S7: Time resolved frequency monitoring of the Ca^{2+} dependent and reversible lipid binding and membrane bridging/linking by the N-terminal deletion mutant AnxA2 Δ14 and alkylated AnxA2 in the QCM-D setup. Numbers illustrate the sequential addition of lipids and proteins: Step 1, SLB formation by SUV adsorption and rupture; step 2, adsorption of the respective AnxA2 derivative; step 3, secondary vesicle application in the form of LUVs; step 4, release of all Ca^{2+}-dependently bound material by chelation with EGTA. The dotted red line marks a reduction in the flow rate from 80.4 µl/min to 8.4 µl/min before the second membrane surface was administered in the form of LUVs. Representative examples of n=2 independent measurements are shown.

Figure S8

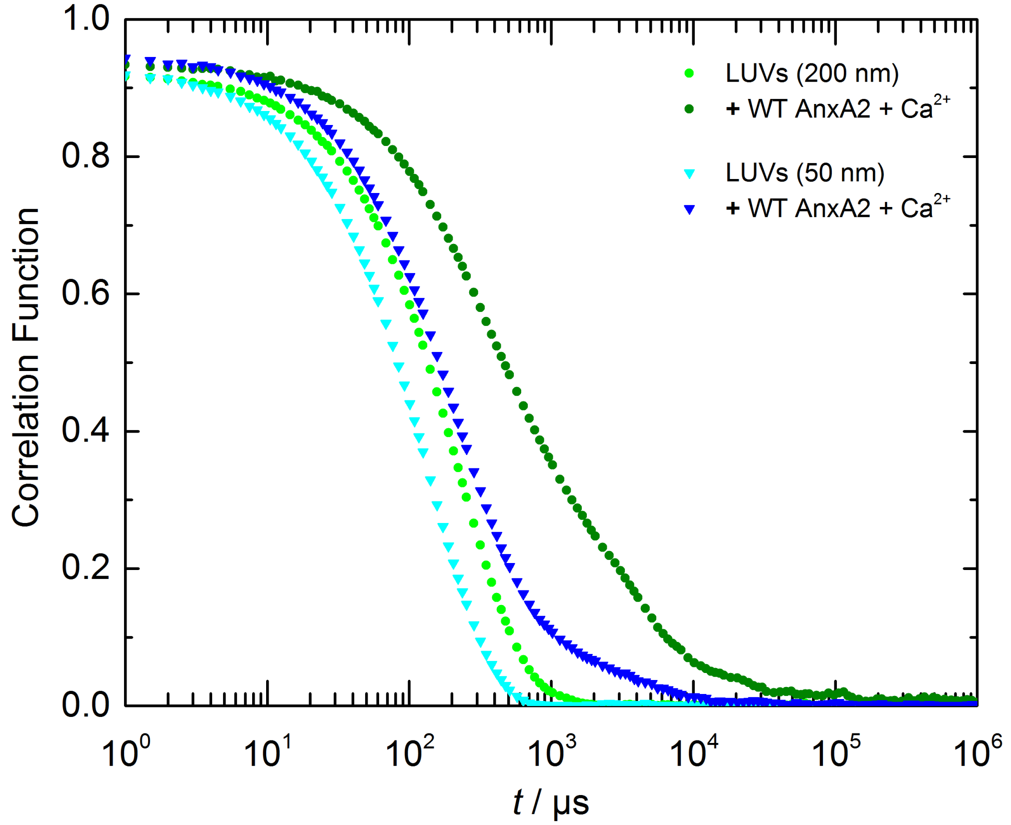

Figure S8: Correlation functions of the DLS measurements shown figure 5B (ii).
